# Substantial clinical benefit, responsiveness, and sensitivity to change of three common outcome measures following shoulder arthroplasty

**DOI:** 10.1177/2050312120946218

**Published:** 2020-07-27

**Authors:** Helen Razmjou, Leila Rahnama, Richard Holtby, Darren Drosdowech, Robin Richards

**Affiliations:** 1 Department of Rehabilitation, Holland Orthopaedic & Arthritic Centre, Sunnybrook Health Sciences Centre, Toronto, ON, Canada; 2 Department of Physical Therapy, Faculty of Medicine, University of Toronto, Toronto, ON, Canada; 3 Sunnybrook Research Institute, Sunnybrook Health Sciences Centre, Toronto, ON, Canada; 4 Department of Exercise Science and Sport Management, Kennesaw State University, Kennesaw, GA, USA; 5 Division of Orthopaedic Surgery, Department of Surgery, Sunnybrook Health Sciences Centre, Toronto, ON, Canada; 6 Division of Orthopaedic Surgery, Department of Surgery, Faculty of Medicine, University of Toronto, Toronto, ON, Canada; 7 Roth McFarlane Hand and Upper Limb Centre, St. Joseph’s Health Care, London, ON, Canada; 8 Division of Orthopaedic Surgery, Western University, London, ON, Canada

**Keywords:** Shoulder arthroplasty, disability, substantial clinical benefit

## Abstract

**Objectives:**

It is important for clinicians involved in the care of patients with advanced glenohumeral osteoarthritis to determine clinically significant change when using outcome measures. There is little information on the amount of substantial clinical benefit in shoulder outcomes after shoulder arthroplasty. The purpose of this study was twofold: (1) to quantify substantial clinical benefit for the American Shoulder and Elbow Surgery score, the Constant Murley Score, and the Western Ontario Osteoarthritis of the Shoulder index and (2) to provide estimates of responsiveness and sensitivity to change for these measures following shoulder arthroplasty.

**Methods:**

The study involved a secondary analysis of previously collected data. The substantial clinical benefit and responsiveness of the measures were calculated based on external anchors related to change in pain, range of motion, and ability to carry out activities of daily living. The areas under curve and standardized response mean represented responsiveness and sensitivity to change.

**Results:**

The data of 159 and 131 patients with complete follow-up at 6 months and 2 years were reviewed. The amount of substantial clinical benefit was dependent on the outcome measure and the external anchor and increased for all measures from 6 months to 2 years. Responsiveness was high (areas under curve > 0.80) at 6 months and further improved at 2 years (areas under curve > 0.88). The standardized response mean values of both time points were over 2.00, indicating high effect sizes. The standardized response means of the Constant Murley Score were statistically significantly higher than the standardized response means of the American Shoulder and Elbow Surgery and Western Ontario Osteoarthritis of the Shoulder.

**Conclusion:**

Amount of substantial clinical improvement in pain, range of motion, and activities of daily living following shoulder arthroplasty depends on the type of outcome measure used. All three measures, the American Shoulder and Elbow Surgery, absolute and relative Constant Murley Score, and Western Ontario Osteoarthritis of the Shoulder, demonstrated good to excellent accuracy and optimal standardized response means.

**Level of evidence:**

Level III, Retrospective Cohort study

## Introduction

Primary glenohumeral joint osteoarthritis is a debilitating condition with progressive stiffness and episodic pain.^1,2^ Shoulder arthroplasty is considered to be an effective treatment for glenohumeral osteoarthritis. The design, functionality, and complication rate of shoulder prostheses^3–6^ have significantly improved over the recent years. The type of arthroplasty performed and the pattern of recovery following surgery are affected by the integrity of rotator cuff muscles and the condition of the glenoid and humeral head.^7–11^ Most patients experience a significantly reduced pain and improved range of motion (ROM) and ability to conduct activities of daily living (ADL) following shoulder arthroplasty.^12,13^

Sensitivity to change is one of the instrument’s properties that measures the ability of the tool to detect change over time.^14^ Although informative, sensitivity to change is insufficient by itself, because it does not take into consideration patient’s values.^15^

Responsiveness^16^ is another psychometric property of an instrument and is defined as the ability of the tool to measure meaningful and important change in clinical state. Respon-siveness is always relative, comparing one scale to another. In relation to measuring meaningful change, the concept of minimal clinically important difference (MCID) was developed in late 1980s.^17^ The MCID defines the minimum improvement threshold which patients perceive as beneficial and which would mandate, in the absence of troublesome side effects and excessive cost, a change in the patient’s management.^17^ In contrast with MCID, which defines the minimum improvement threshold, the substantial clinical benefit (SCB), introduced by Glassman et al.,^18^ is defined as substantial improvement in clinical state as perceived by the patient. Glassman et al.^18^ felt that surgical results should not just meet minimal clinical improvement and rather should exceed that level. Thus, SCB reflects optimal clinical improvement which probably should be the target of orthopedic surgeries, which are performed to optimize the quality of life (QOL). Elective orthopedic surgeries are scheduled in advanced and are performed to improve the health-related QOL as opposed to addressing a medical emergency. These surgeries are more expensive, are associated with more financial stress depending on insurance coverage, and require better justification for the cost. Utilizing the SCB metric values after shoulder arthroplasty helps to identify the denominator of a cost-to-benefit ratio for the appropriateness of performing a costly surgery.^19^

Patient-oriented outcome measures such as the American Shoulder and Elbow Surgery (ASES) score,^20^ the relative Constant Murley Score (CMS),^21^ and the Western Ontario Osteoarthritis of the Shoulder (WOOS) index^22^ are frequently used to assess clinical outcome following shoulder arthroplasty. The ASES and CMS have shown good psychometric properties in this population with a significant body of knowledge on their MCID.^23–28^ There is less information on psychometric properties of the WOOS, and although it has shown statistical change over time, this is mainly affected by large sample sizes.^29–33^ There is only one study that has examined its value in representing individual patient’s points of view in the form of responsiveness.^26^ In a recent systematic review of MCID studies, no studies were identified for the WOOS scale,^34^ making this scale the least examined patient-oriented shoulder outcome measure. In addition, information on the SCB in shoulder arthroplasty remains limited for the ASES and CMS^19,24^ and non-existent for the WOOS. Further assessment of the psychometric properties of these instruments will help to better evaluate the success rate and appropriateness of a costly elective surgery.^19^ The purpose of this study was twofold: (1) to quantify the SCB for the ASES, CMS, and WOOS and (2) to provide estimates of responsiveness and sensitivity to change for these measures following shoulder arthroplasty.

## Materials and methods

The study involved secondary analysis of prospectively collected data of patients with advanced osteoarthritis of the glenohumeral joint who had undergone shoulder arthroplasty and had participated in prior prospective studies.^7,29^ The database included information on demographics, surgical interventions, and pre- and post-operative outcome measures. The outcome measures included in the database were one disease-specific and two joint-specific measures. Approval for use of existing data was obtained from the Sunnybrook Health Sciences Centre.

### Subjects

The inclusion criteria for this study were (1) presence of advanced osteoarthritis with or without rotator cuff pathology that required shoulder replacement including total shoulder arthroplasty (TSA), humeral head replacement (HHR), or reverse shoulder arthroplasty (RSA) and (2) complete information of the follow-up visit at 6 months or 2 years following surgery. The database exclusion criteria were inability to speak or read English, evidence of infection, underlying metabolic disease, avascular necrosis, or capsulorraphy arthropathy. The 6-month and 2-year timeframes were chosen based on literature that reports the most significant change following arthroplasty occurring at 6 months^7^ and the highest reoperation rate occurring within 2 years (63%) with no identifiable peak occurrence after that (average rate 1.1% per year).^35^

### Outcome measures

All patients completed three questionnaires, the ASES,^20^ the CMS,^21^ and the WOOS,^22^ 2–3 weeks prior to surgery. They were given the same outcome measures and an additional global rating and satisfaction survey at the time of follow-up.

The ASES is a self-report 100-point shoulder-specific scale, 50 points of which are derived from patient self-report of pain on a visual analog scale and 50 points of which are computed from a formula using the cumulative score of 10 ADL derived using a 4-point ordinal scale. The ASES was approved by the American Shoulder and Elbow Committee in 1994. The minimum (worst possible) score of the ASES is 0 with a maximum of 100 representing the best functional level.^20^

The CMS is distinguished from other outcome measures which are primarily self-reports by incorporating the clinician’s assessment into the total score. The self-report component of the CMS includes pain and difficulty in ADL, work, sports, and sleep and accounts for 35% of the total score. The objective component incorporates the ROM and strength, accounting for 65% of the total score. One unique feature of the CMS is the ability of this tool to account for age- and sex-related changes by converting the absolute score to the relative score. For the purpose of this study, both absolute CMS (ACMS) and relative CMS (RCMS) scores were provided to evaluate whether normalized values are more informative than the absolute scores. A comprehensive summary of these two measures has been provided in a review by Angst et al.^36^

The WOOS is a self-report disease-specific QOL outcome measure with 19 questions presented in four domains (physical symptoms, life style, sports/work, and emotions). The total score is the summated score of these four domains with a maximum of 1900 (worst possible raw score). The aggregate score is then subtracted from 1900 and divided by 19 to provide a percentage with 0% being the worst and 100% being the best possible score.^22^

The ASES and CMS have established validity and reliability^37,38^ and responsiveness^25,26^ in patients with glenohumeral osteoarthritis, and the SCB following shoulder arthroplasty has been reported for the ASES^19,24^ and the CMS.^19^ There is less information on psychometric properties of the WOOS in the English language with one study on its responsiveness^26^ and a number of studies reporting statistical change after arthroplasty surgery.^29–33^

The global rating and satisfaction survey addressed seven questions with respect to patients’ expectations being met in domains including achieving pain relief, improved ROM and ability to perform ADL (Supplemental Appendix A). For the purpose of this study, we used the answers to these questions as external anchors and reported on the overall satisfaction on a Likert-type scale.

### Statistical analyses

We estimated our sample size based on the ability of the measures to detect a substantial clinical change based on a small effect size of 0.2 in one group over time. The lowest SCB change reported in the literature is 19 points for the CMS.^19^ By choosing an estimated standard deviation of 10 and a small effect size of 0.2,^39^ using the below formula for one group (paired t-test), a minimum of 43 patients with complete data (N ={(Za–Zb)SD/d}^2^ where a = 0.05, Za = 1.65, b = 0.20, Zb = 0.84, SD = 2, d = 0.2 × 19 = 3.8, N ={(1.65 + 0.84) 10/3.8}^2^ = 43) is required to detect substantial change over time. Descriptive analyses of patient characteristics and initial outcome measure summary scores of a convenient sample were performed and compared between 6 months and 2 years. Change over time was examined in the ASES, CMS, and WOOS at both time points using t-test statistics.

In this study, the SCB and responsiveness were calculated using an anchor-based approach^17^ and the mean change method.^40^ In anchor-based methodology, the patient’s overall impression of improvement is captured by a global rating. At the time of follow-up, the global anchor questions documented improvement in pain, ROM and ability to carry out the ADL. The content of the anchor was construct-specific; physical symptoms (WOOS) and pain (ASES and CMS) were correlated with external anchor of pain. The lifestyle domain of the WOOS that has questions about daily activities and the ADL domains of the ASES and CMS were correlated with the anchor of ADL. The ROM of the CMS was correlated with the ROM anchor. All anchors were correlated with the total scores of the outcome measures. A minimum of 0.30 as suggested by Revicki et al.^41^ was adopted as a correlation threshold to define an acceptable association between the anchor and the change score.

### Operational definition

In this study, substantial improvement was defined as the highest level of recovery in the respected category, labeled as “a lot improved” in the survey (Supplemental Appendix A). The SCB was obtained by subtracting the mean change of categories of “no improvement” or “just a little bit improved” from the category of “a lot improved.” Patients with “somewhat improvement” were deleted from the analysis as this group did not meet the substantial threshold for SCB. This is consistent with the only study on SCB of the shoulder instruments which used the category of “much better” as opposed to “better” as a criterion for substantial change.^19^

For responsiveness, we constructed the receiver operator characteristic (ROC) curves with true-positive rate (sensitivity) on the y-axis and false-positive rate (1—specificity) on the x-axis using the same dichotomized anchor-based question as the external criterion and calculated the area under the curve (AUC) to examine the predictive ability and the overall responsiveness of the instruments.^42^ Similar to a diagnostic test, an instrument that classifies patients correctly as improved versus not improved has a larger area under the ROC curves. The AUC curves provide more insight into the relationship between change measured with an instrument versus an external criterion for an improvement.^15^ An AUC of 1.0 represents perfect differentiation by the instrument with 100% sensitivity and specificity, an AUC of 0.90 and higher indicates excellent accuracy, and an AUC of 0.80 and higher indicates good accuracy.

Sensitivity to change measures the magnitude of change statistically and was based on the standardized response mean (SRM) calculated as the ratio between the mean change score and the standard deviation of the change score.^14^ The SRM expresses change scores in terms of the underlying sampling distribution and is a standardized indicator of power of an instrument to detect true change, with larger values indicating higher sensitivity to change.^15^ Cohen’s criteria were used to interpret the magnitude of SRM values.^43^

## Results

Data of 168 patients who had arthroplasty surgery were reviewed. Three patients had died of natural causes. Six patients had missing data on the satisfaction questionnaire. A total of 159 patients had complete data prior to surgery and at the 6-month follow-up. In all, 12 patients had missed the 2-year follow-up, with 131 patients having complete data at both 6 months and 2 years. Table 1 shows the number and percentage of the types of surgeries in each cohort. Patients with primary osteoarthritis with an intact rotator cuff received an anatomical TSA (84% and 86% in the 6-month and 2-year cohorts, respectively). The HHR surgery was performed for patients with primary osteoarthritis with a deficient glenoid bone, inflammatory arthritis, or humeral head fractures (10% and 9%, respectively). RSA was performed for cuff tear arthropathy where anatomical arthroplasty was not a viable option due to excessive abnormal loading of deltoid in the absence of rotator cuff and superior tipping of the glenoid component (6% and 5%, respectively). There were no statistically significant differences (p > 0.05) between the 6-month and 2-year samples in age, sex distribution, dominate side, affected side, type of surgery, or pre-operative scores of the ASES, CMS, or WOOS, indicating the samples were comparable (Table 1).

**Table 1.** Demographics and pre-operative outcomes between cohorts.

Demographics	Cohort at 6 months (N = 159)	Cohort at 24 months (N = 131)	Statistics
Age (mean, SD)	68 (8)	68 (8)	t-test = 0.46, p = 0.64
Sex (N, %)			
Male	64 (60%)	53 (60%)	χ^2^ = 0.001, p = 0.97
Female	95 (40%)	78 (40%)	
Dominant side			
Right	141 (89%)	118 (91%)	FET = 0.049, p = 0.89
Left	15 (9%)	10 (8%)	
Ambidextrous	3 (2%)	2 (1%)	
Side of surgery			
Right	88 (55%)	71 (54%)	χ^2^ = 0.04, p = 0.84
Left	71 (45%)	60 (46%)	
Pre-operative outcome measures			
ASES	30.1 (16)	31.8 (17)	t-test = 0.91, p = 0.36
RCMS	27.0 (15)	27.7 (15)	t-test = 0.40, p = 0.69
WOOS	26.0 (15)	26.9 (17)	t-test = 0.78, p = 0.44
Type of arthroplasty			
TSR	133 (84%)	113 (86%)	
HHR	16 (10%)	12 (9%)	χ^2^ = 0.50, p = 0.78
RSA	10 (6%)	6 (5%)	

SD: standard deviation; ASES: American Shoulder and Elbow Surgeons; RCMS: Relative Constant Murley Score; WOOS: Western Ontario Osteoarthritis of the Shoulder; TSR: Total Shoulder Arthroplasty; HHR: Humeral Head Replacement; RSA: Reverse Shoulder Arthroplasty; FET: Fisher’s Exact Test.

Patients showed improvement in ASES, CMS, and WOOS scores at 6 months (p < 0.0001) and at 2 years following surgery (p < 0.0001) with a statistically significant difference between 6 months and 2 years (p < 0.0001). Table 2 shows pre- and final post-operative scores of the total and sub-domain of each instrument at 2 years. Table 3 summarizes the construct domains of the outcome measures and the corresponding transition item anchors.

**Table 2.** Pre-operative and 2-year post-operative scores of all outcome measures with their subdomains.

Instrument	Items (range)	Pre-op scores Mean (SD)	Post-op scores Mean (SD)
WOOS			
Symptoms	6 (0–600)	425.85 (112)	98.54 (130)
Sports/work	5 (0–500)	389.93 (82)	115.15 (137)
Life style	5 (0–500)	380.20 (87)	104.56 (131)
Emotions	3 (0–300)	192.20 (76)	45.66 (74)
Total	19 (100)	26.90 (17)	80.89 (24)
ASES			
Pain	1 (1–10)	6.24 (2.56)	1.19 (2)
ADL	10 (0–30)	7.84 (4.63)	22.57 (9)
Total	11 (0–100)	31.80 (17)	80.67 (19)
CMS			
Pain	1 (0–15)	1.61 (3)	11.56 (4)
ADL	2 (0–20)	6.69 (3)	14.81 (4)
ROM	4 (0–40)	8.56 (6)	28.67 (9)
Power	1 (0–25 lb)	3.97 (4)	9.41 (4)
Total ACMS	10 (0–100^+^)	21.03 (12)	64.56 (18)
Total RCMS	10 (0–100^+^)	27.70 (15)	80.67 (19)

SD: standard deviation; WOOS: Western Ontario Osteoarthritis of the Shoulder; ASES: American Shoulder and Elbow Surgery; ADL: Activities of Daily Living; CMS: Constant Murley Score; ROM: Range of Motion; ACMS: Absolute Constant Murley Score; RCMS: Relative Constant Murley Score.

**Table 3.** Descriptive score data before and 2 years after surgery for transition items of the correspondent anchors.

Outcome measure	Transition group No/a little bit		Transition group “A lot better”	
Anchor	Pre-op scores	Post-op scores	Pre-op scores	Post-op scores
	Mean (SD)	Mean (SD)	Mean (SD)	Mean (SD)
WOOS				
Physical symptoms^a^				
Pain	465.85 (104)	398 (90)	423.32 (115)	63.76 (84)
Life style^b^				
ADL	407.27 (59)	345.90 (117)	373.56 (80)	61.89 (83)
Total WOOS%				
Pain	19.14 (14)	25.71 (11)	27.33 (16)	87.23 (16)
ADL	21.63 (12)	32.92 (23)	28.73 (16)	89.28 (12)
ROM	19.64 (`5)	35.57 (24)	28.15 (16)	89.55 (14)
ASES				
Pain^a^				
Pain	6.28 (3)	6.00 (2)	6.15 (3)	0.70 (1)
ADL^b^				
ADL	6.81 (3)	11.36 (6)	7.94 (5)	24.08 (5)
Total ASES				
Pain	29.76 (19)	34.04 (9)	31.33 (17)	85.66 (13)
ADL	28.189 (16)	46.21 (20)	33.24 (16)	86.92 (12)
ROM	29.52 (16)	49.52 (19)	32.82 (16)	87.02 (11)
CMS				
Pain^a^				
Pain	0.71 (3)	2.85 (3)	1.69 (3)	12.52 (4)
ADL^b^				
ADL	5.81 (2)	7.45 (4)	7.07 (3)	24.08 (5)
ROM^c^				
ROM	6.92 (5)	15.85 (9)	9.11 (6)	31.56 (6)
Total ACMS				
Pain	14.00 (7)	23.42 (12)	21.76 (12)	68.78 (13)
ADL	16.72 (7)	34.00 (19)	22.51 (12)	70.67 (13)
ROM	16.23 (8)	33.61 (15)	22.12 (14)	71.09 (12)
Total RCMS				
Pain	18.91 (9)	33.14 (17)	28.70 (15)	93.69 (18)
ADL	22.02 (9)	45.81 (25)	29.61 (15)	96.03 (15)
ROM	21.52 (11)	47.08 (23)	22.13 (12)	71.09 (12)

SD: standard deviation; WOOS: Western Ontario Osteoarthritis of the Shoulder; ADL: Activities of Daily Living; ROM: Range of Motion; ASES: American Shoulder and Elbow Surgery; CMS: Constant Murley Score; ACMS: Absolute Constant Murley Score; RCMS: Relative Constant Murley Score.

Number of transition categories of “no/a little bit” and “a lot better” after removing “somewhat better”: Pain domain (7/113), ADL (11/99), and ROM (14/99). Total scores of WOOS, ASES, and CMS were correlated with all three transition items of pain, ADL, and ROM anchors.

a Symptoms and pain were matched with the pain anchor.

b Lifestyle domain of the WOOS and ADL domains of ASES and CMS were matched with the ADL anchor.

c ROM domain of the CMS was matched with the ROM anchor.

All correlations between the corresponding anchor and the change score were above 0.30.

Table 4 demonstrates the SCB, AUC, and SRM values for the anchors of pain, ADL, and ROM for both follow-ups. For the external anchor of pain, the SCB was 31.8, 22.3, 29.1, and 39.5 for the ASES, ACMS, RCMS, and WOOS, respectively, at 6 months. The pain-related SCB increased to 48.9, 37.6, 50.6, and 53.3 for the ASES, ACMS, RCMS, and WOOS, respectively, at 2 years. The SCB for external anchors of ROM and ADL is shown in Table 4 and show the same pattern of increase over time. There was an approximate 10-point increase in the SCB of the RCMS versus ACMS, which is the result of adjustment for age and sex in this population. The SCB calculated for the RCMS, however, fell within the range of other measures and did not necessarily overestimate function as indicated by Yian et al.^44^

**Table 4.** Substantial clinical benefit (SCB), responsiveness, and sensitivity to change.

Outcome measure	SCB Mean ± SD (95% CI)	Responsiveness AUC (95% CI)	Sensitivity to change SRM (95% CI)
6-month follow-up			
ASES			
Pain	31.8 ± 20.9 (15.7–47.8)	0.80 (0.59–1.00)	
ROM	28.5 ± 20.1 (18.1–38.9)	0.81 (0.69–0.92)	2.00 (1.83–2.15)
ADL	29.7 ± 21.2 (17.29–42.13)	0.83 (0.69–0.97)	
ACMS			
Pain	22.3 ± 14.3 (9.8–34.2)	0.87 (0.59–1.00)	
ROM	23.0 ± 19.2 (15.5–30.4)	0.89 (0.83–0.96)	2.21 (2.03-2.38)
ADL	19.7 ± 14.5 (10.6–28.7)	0.84 (0.74–0.95)	
RCMS			
Pain	29.1 ± 20.2 (12.3–45.9)	0.85 (0.70–1.00)	
ROM	29.8 ± 19.2 (19.2–40.4)	0.87 (0.78–0.96)	2.25 (2.13–2.60)
ADL	26.5 ± 20.0 (13.1–40.0)	0.83 (0.72–0.94)	
WOOS			
Pain	39.5 ± 19.9 (24.08–54.83)	0.92 (0.85–0.99)	
ROM	34.4 ± 19.3 (24.41–44.4)	0.89 (0.81–0.97)	2.24 (2.08–2.40)
ADL	34.8 ± 19.0 (23.7–45.9)	0.91 (0.82–0.99)	
2-year follow-up			
ASES			
Pain	48.9 ± 18.6 (34.6–63.3)	0.97 (0.95–1.00)	
ROM	34.2 ± 19.2 (23.3–45.0)	0.88 (0.76–0.99)	2.17 (2.00–2.34)
ADL	35.5 ± 19.8 (23.1–48.0)	0.86 (0.70–1.00)	
ACMS			
Pain	37.6 ± 25.9 (28.4–46.7)	0.98 (0.96–1.00)	
ROM	31.5 ± 14.5 (23.5–39.4)	0.95 (0.90–1.00)	2.49 (2.31–2.66)
ADL	30.7 ± 15.2 (19.6–41.7)	0.92 (0.81–1.00)	
RCMS			
Pain	50.6 ± 21.5 (34.0–67.3)	0.98 (0.94–1.00)	
ROM	41.6 ± 21.2 (29.1–54.0)	0.93 (0.85–1.00)	2.43 (2.24–2.60)
ADL	42.3 ± 21.7 (28.6–56.0)	0.92 (0.81–1.00)	
WOOS			
Pain	53.3 ± 19.6 (38.2–68.5)	0.97 (0.95–1.00)	
ROM	45.5 ± 20.1 (34.1–56.9)	0.91 (0.77–1.00)	2.12 (1.95–2.30)
ADL	50.1 ± 19.5 (37.8–62.4)	0.92 (0.79–1.00)	

SCB: substantial clinical benefit; SD: standard deviation; CI: confidence interval; AUC: areas under curve; ASES: American Shoulder and Elbow Surgery; ROM: Range of Motion; ADL: Activities of Daily Living; ACMS: Absolute Constant Murley Score; RCMS: Relative Constant Murley Score; WOOS: Western Ontario Osteoarthritis of the Shoulder; SRM: standardized response mean; CMS: Constant Murley Score.

At 2 years, there was a statistically significant difference between the SRM of the WOOS and ACMS (p = 0.014) and RCMS (p = 0.004) in favor of the CMS. There was a statistically significant difference between the SRM of the ASES and ACMS (p = 0.04) and RCMS (p = 0.01) in favor of the CMS. There was no statistically significant difference between the ASES and WOOS (p = 0.68).

In summary, to achieve substantial improvement at 2 years following shoulder arthroplasty, approximately 50% change in the scores of the ASES, RCMS, and WOOS is necessary.

Based on the defined SCB for pain, 78 (60%) of the patients achieved substantial improvements in pain based on the ACMS, RCMS, and WOOS scores at 2 years, which is considered a minimum time frame for stable outcomes after arthroplasty. Achieving substantial improvement in ADL varied from 85 (65%) for WOOS, 92 (70%) for ACMS, 95 (73%) for RCMS, and 95 (73%) for ASES. Finally, 90 (69%), 92 (70%), 95 (73%), and 98 (75%) of the patients achieved substantial improvement in their ROM based on the ACMS, WOOS, RCMS, and ASES scores, respectively.

The AUC values ranged from 0.80 to 0.91 at 6 months and showed improvement at 2 years, ranging from 0.86 to 0.98. The AUC values of the ACMS were slightly higher than those of the RCMS. The SRM values of all three measures were over 2.0 at both follow-ups, representing large effect sizes (Table 4). Of the 131 patients in the 2-year cohort, 106 (81%) reported to be very satisfied, while 5 (4%) reported significant dissatisfaction. Fourteen (11%) reported a little bit satisfied with 6 (5%) reporting a little bit dissatisfied.

The AUC values for pain were >0.90 at 2 years for ASES, ACM, RCM, and WOOS, indicating excellent accuracy with slightly smaller values for the ASES for ADL (0.88) and ROM (0.86). All SRM values were 2.00 or more, indicating optimal sensitivity to change for all three outcomes.^39^ At 2 years, the SRM values of both ACMS and RCMS were superior to ASES (p = 0.04 and p = 0.01) and WOOS (p = 0.014, p = 0.004), respectively. There was no statistically significant difference between the SRM values of the ASES and WOOS (p = 0.68) (Table 4).

## Discussion

We observed an overall improvement in the ASES, absolute and relative CMS, and WOOS scores at 6 months and 2 years following shoulder arthroplasty, which is consistent with previous studies.^7,12,13^ The SCB values for pain were higher than the SCB for the ADL and ROM across all outcome measures, indicating higher levels of improvement in the ASES, CMS, and WOOS were required for the patients to report substantial pain relief. As a result, fewer patients (approximately 60%) reported substantial improvement in their pain as compared with approximately 70% reporting substantial improvement in their ADL and ROM. This shows that pain remains the most challenging problem after shoulder arthroplasty. The lack of full relief of pain following shoulder arthroplasty has been previously reported^45–48^ and may explain higher SCB scores associated with pain relief than the SCBs associated with improvement in ROM and functional abilities.

Two previous studies have examined the SCB of the ASES in relation to activity and overall improvement. Werner et al.^24^ subtracted the mean change of ASES score of those reporting “no change” and “somewhat dissatisfied” from the mean change of ASES score of those who had reported “very satisfied” and measured the SCB associated with the overall satisfaction with surgery (SCB = 37.4), ability to do housework/yardwork (SCB = 21.6), and recreational activities (SCB = 19.2) at 2 years post arthroplasty. Simovitch et al.^19^ calculated the SCB as the mean difference between the unchanged group “no change/worse” and the changed group “much better” and reported SCB values of 31.5 and 19.1 for substantial overall satisfaction in the ASES and CMS, respectively, at the minimum of 2 years. The results of the SCB values of the ASES by the previous studies are more consistent with our findings at 6 months. We had higher SCB values at 2 years post-operatively, particularly for the CMS and WOOS. These discrepancies may in part be related to differences in patient population, the external anchor scales, and the variables they represented (e.g. pain vs. overall satisfaction). In addition, these measures have more item variability than the ASES, which may explain the higher SCB values. The relative CMS scores were clearly higher than the absolute values and had a slightly lower responsiveness based on the AUC curves and lower sensitivity to change based on the SRM values. However, since the adjusted values were similar to the WOOS and ASES SCBs, there is no harm in using the relative CMS.

We found AUC values of > 0.80 at 6 months and > 0.88 at 2 years for all outcomes. In orthopedic surgery, a minimum of 2-year follow-up is required to establish reliable results for any type of arthroplasty, and the AUC values at 2 years for all three outcome measures met the criteria for good to excellent accuracy. There is some information in the literature on the responsiveness of ASES and CMS based on AUC values. Angst et al.^25^ have reported AUC values of 0.76 and 0.77 for the ASES and CMS, respectively. The authors used a global rating of change as their external anchor and compared the “slightly better” to “much better.” This may explain their lower AUC values as the difference between “no/minimal improvement” and “a lot better” used in this study is expected to be higher.^25^

In this study, all three measures produced SRM > 2, which indicates optimal sensitivity to change. Similar to our findings, the sensitivity to change using SRM values of the ASES and CMS are reported to be > 0.80 in the literature.^22,25,49^ The SRM for the ASES has a wide range of 0.93,^49^ 1.3,^22^ and 2.13.^25^ The SRMs for the CMS have varied from 1.2^22^ to 2.23.^25^ The SRM of the WOOS in the development study by Lo et al.^22^ is reported to be 1.91. Our results are more consistent with Angst et al.’s^25^ study. The SRM values of the ACMS and RCMS were statistically superior to the WOOS and ASES at 2 years, which may suggest that a combined self-report and clinician outcome (e.g. CMS) is more sensitive to change than pure self-report outcomes after shoulder arthroplasty.

To our knowledge, this is the first study that has examined the SCB of the WOOS. Our results add to the body of literature in two areas: by providing an estimated level of substantial improvement and by providing further evidence that using multi-page disease-specific QOL measures may not add valuable clinical information beyond what is learned by the shorter joint-specific measures. Future comparative studies are needed to further assess the response burden of these outcome measures.

The WOOS was developed as a disease-specific QOL outcome measure for patients with osteoarthritis.^22^ A properly designed QOL measure is expected to be constrained to *indicator variables*.^50^ Fayers^50^ defines *indicator variables* (e.g. depression) as facets of the QOL whose value depends solely upon the QOL construct. A large number of WOOS items represent symptoms and difficulty performing certain tasks that are *causal indicators* of the QOL.^50^ The *causal indicators* (e.g. pain and inability to wash the hair) are variables that have a causal relationship with the QOL and have impact upon patients but are not a true representative of QOL. While pain affects QOL, not all patients with low QOL have pain and not all patients with pain have low QOL. Pain and dysfunction are indicators of the status of musculoskeletal disease and not necessarily a cause of reduced QOL.^50–53^

The WOOS uses a single total score as the representative of change of four different domains (symptoms, sports/recreation/work, life style, emotions) that display different change trajectories. These domains do not recover at the same speed.^54,55^ Although we did not measure the response burden in our study, in general terms, the response burden and the effort both patients and clinicians make to respond to or to calculate the total score of a multi-domain instrument^56^ adds to the challenge of collecting information in busy clinical settings. In addition, an osteoarthritis disease-specific outcome has questionable utility in patients with multiple or overlapping conditions such as cuff tear arthropathy or instability-related osteoarthritis.

In this study, we did not separate different types of arthroplasty due to a small number of HHR and RSA. While Werner et al.^24^ reported no difference in SCB values between the TSA and RSA, Simovitch et al.^19^ reported higher values for TSA than RSA. Since superiority in outcome in favor of TSA has been well-established,^10,11,57–63^ slightly lower SCB scores are expected in the presence of associated rotator cuff or bony morbidity seen in patients who had undergone the HHR and RSA surgeries.

Our study has the following limitations: it involved secondary analysis of prospectively collected data of patients operated by three different orthopedic surgeons in an academic center, which may limit the generalizability of our findings. The information on education and co-morbidity was not available for all patients and was not included in the analysis. For establishing statistical equivalence or lack of inferiority among instruments, larger sample sizes are required and future studies are needed to validate our findings. Nevertheless, this study provides preliminary data that the AUC and SRM values of these measures meet high standards for accuracy.

## Conclusion

The amount of SCB in pain, ADL, and ROM after arthroplasty depends on the outcome measure used. All three measures studied here, the ASES, CMS, and WOOS, demonstrated good to excellent responsiveness and optimal sensitivity to change. Approximately 70% of the patients achieved substantial improvement in ROM and ADL. The substantial pain relief was achieved in 60% of the patients.

## Supplemental Material

Appendix_A – Supplemental material for Substantial clinical benefit, responsiveness, and sensitivity to change of three common outcome measures following shoulder arthroplasty Click here for additional data file. Supplemental material, Appendix_A for Substantial clinical benefit, responsiveness, and sensitivity to change of three common outcome measures following shoulder arthroplasty by Helen Razmjou, Leila Rahnama, Richard Holtby, Darren Drosdowech and Robin Richards in SAGE Open Medicine
