# Supplementary material for: Substantial clinical benefit, responsiveness, and sensitivity to change of three common outcome measures following shoulder arthroplasty
Source: SAGE Open Med. 2020 Jul 27;8:2050312120946218. doi: 10.1177/2050312120946218 (PMC7385837; doi:10.1177/2050312120946218)
Supplement: Appendix_A – Supplemental material for Substantial clinical benefit, responsiveness, and sensitivity to change of three common outcome measures following shoulder arthroplasty [file Appendix_A.pdf]

POST OPERATIVE EXPECTATION QUESTIONNAIRE

1. Has your surgery helped with **pain relief**?

- ❑ 1 no, surgery did not help with my pain
- ❑ 2 yes, but just a little
- ❑ 3 yes, somewhat
- ❑ 4 yes, a lot

2. Has your surgery increased your painfree **range of motion**?

- ❑ 1 no, surgery did not improve my range of motion
- ❑ 2 yes, but just a little
- ❑ 3 yes, somewhat
- ❑ 4 yes, a lot

3. Has your surgery improved your **ability to carry out the normal activities of daily living**?

- ❑ 1 no, surgery did not improve my ability to carry out the normal daily activities
- ❑ 2 yes, but just a little
- ❑ 3 yes, somewhat
- ❑ 4 yes, a lot

4. Has your surgery improved your **ability to care for others**?

- ❑ 1 no, surgery did not improve my ability to interact and care for others
- ❑ 2 yes, but just a little
- ❑ 3 yes, somewhat
- ❑ 4 yes, a lot
- ❑ 5 not applicable

5. Did you **return to work** following your surgery?

- ❑ 1 no, I am not working
- ❑ 2 yes, light/modified work
- ❑ 3 yes, full capacity/regular duties
- ❑ 5 not applicable

6. Are you able to participate in **the leisure**, **sports or recreational activities** you did before your problem started?

- ❑ 1 no, I cannot participate in sports/recreational activities.
- ❑ 2 yes, but not as much as before
- ❑ 3 yes, as much as before
- ❑ 5 not applicable

7. Is the area operated upon, **back to the way it was** before your problem started?

- ❑ 0 no, not at all
- ❑ 1 no, but a little improved
- ❑ 2 no, but somewhat improved
- ❑ 3 yes, completely

8. If you could turn back the clock to prior to your surgery, would you undergo the surgery again?

- ❑ 0 no
- ❑ 1 yes

9. **Finally, how satisfied are you overall with the results of your surgery?**

- ❑ 1 very satisfied
- ❑ 2 somewhat satisfied
- ❑ 3 a little bit satisfied
- ❑ 4 a little bit dissatisfied
- ❑ 5 somewhat dissatisfied
- ❑ 6 very dissatisfied

Thank you for completing this questionnaire!

Copyright © 2020 Sunnybrook Health Sciences Centre. All rights reserved by Sunnybrook Health Sciences Centre, operating as the Holland Orthopaedic & Arthritic Centre.
